# Action Levels for the Prevention of Work-Related Musculoskeletal Disorders in the Neck and Upper Extremities: A Proposal

**DOI:** 10.1093/annweh/wxab012

**Published:** 2021-04-08

**Authors:** Inger Arvidsson, Camilla Dahlqvist, Henrik Enquist, Catarina Nordander

**Affiliations:** Division of Occupational and Environmental Medicine, Department of Laboratory Medicine, Lund University, Medicon Village, SE-223 81, Lund, Sweden

**Keywords:** exposure assessment, exposure-response relationships, risk assessment, technical measurements, threshold limit values

## Abstract

There are several well-known risk factors for work-related musculoskeletal disorders (MSDs). Despite this knowledge, too many people still work in harmful conditions. The absence of occupational exposure limits (OELs) for physical workload impedes both supervision and preventive work. To prevent myalgia, tendon disorders, and nerve entrapments in the upper musculoskeletal system, we propose action levels concerning work postures, movement velocities and muscular loads recorded by wearable equipment. As an example, we propose that wrist velocity should not exceed 20°/s as a median over a working day. This has the potential to reduce the prevalence of carpal tunnel syndrome (CTS) in highly exposed male occupational groups by 93%. By reducing upper arm velocity in highly exposed female groups to the suggested action level 60°/s, the prevalence of pronounced neck/shoulder myalgia with clinical findings (tension neck syndrome) could be reduced by 22%. Furthermore, we propose several other action levels for the physical workload. Our ambition is to start a discussion concerning limits for physical workload, with the long-term goal that OELs shall be introduced in legislation. Obviously, the specific values of the proposed action levels can, and should, be discussed. We hope that quantitative measurements, combined with action levels, will become an integral part of systematic occupational health efforts, enabling reduction and prevention of work-related MSDs.

## Introduction

It has been known for decades that awkward postures, high muscular load or repetitive movements can cause work-related musculoskeletal disorders (MSDs). Despite this knowledge, the risks are still found in many branches, such as elderly care, cleaning, warehousing, and in the manufacturing industry, to name a few. Years of research and efforts by companies, trade unions, occupational health services, the Work Environment Authorities and others, have not solved the problem that too many people get seriously affected of work-related MSDs.

To reduce the risk of work-related illness, there are—for most occupational risks—occupational exposure limits (OELs) that by law must not be exceeded. This applies, for example, to chemicals, noise, and vibrations in the workplace. Because of preventive measures that have been taken to comply with the law, several occupational risks have been reduced (e.g. regarding asbestos, lead, benzene, and noise). In our opinion, there is no practical or fundamental difference between risk assessment of these and that of physical workload. However, despite the large problem with work-related MSDs, there are no OELs for physical workload. Instead, the regulations from, e.g. the Swedish Work Environment Authority governing physical workload are vaguely formulated and based on assessment models, checklists, and observational methods. Generally, observational methods are relatively easy to use, associated with limited costs, and can be suitable tools for obtaining an overview and a rough estimate of loads that occur in the work, especially if the work is characterized by a limited work content. For example, the observational method suggested by the American Conference of Governmental Industrial Hygienist (ACGIH, 2019) is a combined estimation of force requirements and frequency of hand movements, related to a Threshold Limit Value (TLV) for hand-intensive work. However, observational methods suffer from limitations, e.g. as they have been shown to produce significant differences between experts assessing the same task (NIOSH, 1997; NRC, 2001; Eliasson *et al.*, 2017). Furthermore, it is not possible to obtain a specific value of movement velocities and muscle strain by observations alone. Thus, we are convinced that OELs based on quantitative objective methods are needed for a successful prevention of work-related MSDs.

Measuring devices worn by workers allow the physical workload to be recorded throughout the working day. Postures and movements, the muscular activity required to perform the task, and the recovery time, can be recorded by applying sensors and electrodes to the skin. Technical recordings capture movement velocities and muscular load, which are not easily observed (Spielholz *et al.*, 2001; Jones and Kumar, 2010). Furthermore, such methods overcome limitations with poor inter- and intra-observer reliability. Historically, technical recordings have been perceived as complicated, time consuming, and costly. However, there is an ongoing development of simplified and user-friendly methods, and a growing interest among researchers and practitioners to use technical/objective methods as a part of the risk assessment. Therefore, the time is ripe for action levels, or TLVs, to which the results of the recordings can be related.

To be able to define OELs for physical workload, knowledge of the relationship between physical workload and the risk of MSDs is needed. At Occupational and Environmental Medicine in Lund, we have developed methods for technical recordings achieving objective quantitative data of the physical workload, and applied these for more than 1000 recordings on employees in about 60 different occupations (e.g. Hansson *et al.*, 2009, 2010; Arvidsson *et al.*, 2012). We have also mapped the occurrence of MSDs in the studied groups, by questionnaire and by clinical examinations (Nordander *et al.*, 2009; Arvidsson *et al.*, 2016). The rationale for the collection of this kind of data, from many professional groups, was to be able to define quantitative exposure–response relationships between the physical workload and the risk of MSDs in the neck and the upper extremity (Nordander *et al.*, 2013, 2016; Balogh *et al.*, 2019). Several other research-groups have also reported relationships between different aspects of the physical workload and MSDs, for example Veiersted *et al.* (1993); van den Heuvel *et al.* (2006); Takamiya *et al.* (2006); Palmer and Smedley (2007); Van Rijn *et al.* (2009, 2010); Da Costa and Vieira (2010); Mayer *et al.* (2012); Bodin *et al.* (2012); Hanvold *et al.* (2012, 2013); Sjøgaard and Søgaard (2014); Dalbøge *et al.* (2014, 2018); and Lund *et al.* (2019).

We propose action levels for physical workload, based on technical recordings of the exposure. The proposed action levels are based on the studies of our research group, together with knowledge from other scientific literature. If these action levels are exceeded, there is a high risk of MSDs, and a need of preventive actions. It is our intention and hope that such action levels will be implemented in risk assessments and form a basis for discussions on preventive measures, in the same way as OELs are used for other harmful exposures.

## How the action levels were defined

The main basis of the proposed action levels is our research of the relationship between the physical workload and MSDs. A number of cross-sectional studies of different occupational groups were conducted during the period 1987–2016. The studies comprised a wide range of occupations and we ensured a considerable spread in physical workload, although not completely exhaustive. We used the same methods for data collection in all studies, a strategy that has resulted in a large database.

The physical workload was quantified in occupational groups within which the workers/participants performed the same or very similar work tasks. In these groups, recordings were performed during full working days in subsamples, whose mean value was considered to represent the physical workload in the particular type of work. We used s*urface electromyography* to record activity in the trapezius muscles and the forearm muscles. By *inclinometry*, we recorded work postures of the head, upper back, and upper arms, and wrist g*oniometry* to record palmar and dorsal flexion of the wrist. Movement velocities were then calculated by derivation. More information on these methods is given in Nordander *et al.* (2004), Hansson *et al.* (2006), and Balogh *et al.* (2009).

In the groups on which technical recordings were performed, we also carried out an extensive survey of MSDs in the neck, shoulders, arms, and hands, based on self-reporting of complaints (Kourinka *et al*., 1987) and on diagnoses (e.g. rotator cuff syndrome and carpal tunnel syndrome (CTS)) defined by a standardized clinical examination (Nordander *et al.*, 2009; Jonker *et al.*, 2015). The prevalences of complaints and diagnosed disorders were calculated for each occupational group. Assessment of exposure–response relationships were conducted on group-level data; in 27 occupational groups for elbows/hands disorders (Nordander *et al.*, 2013), and in 33 groups for neck/shoulders disorders (Nordander *et al.*, 2016). The study of Balogh *et al.* (2019) included an extended population of in total 51 groups, where personal factors and psychosocial conditions were taken into consideration. In essence, adjustment for these variables did not influence the associations between exposure and outcome.

**Figure 1.**
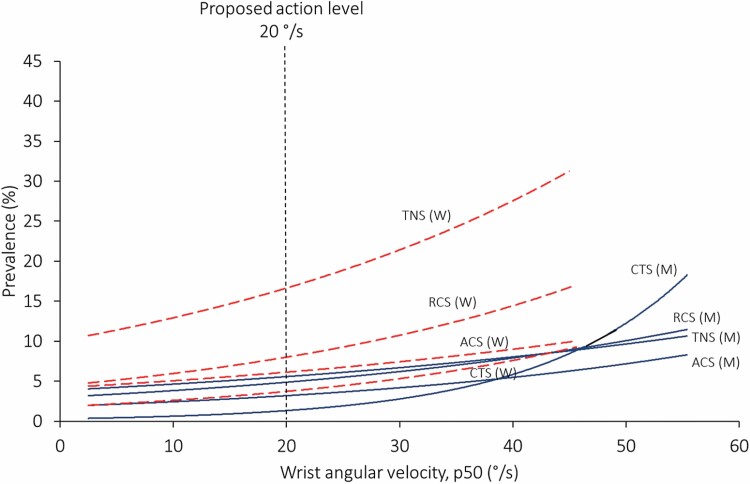
Statistically significant associations between tension neck syndrome (TNS), rotator cuff syndrome (RCS), acromioclavicular syndrome (ACS), and carpal tunnel syndrome (CTS), versus wrist angular velocity, in women (red dotted lines) and men (blue continuous lines), calculated by Poisson regression. Modified from Balogh *et al.* (2019).

**Figure 2.**
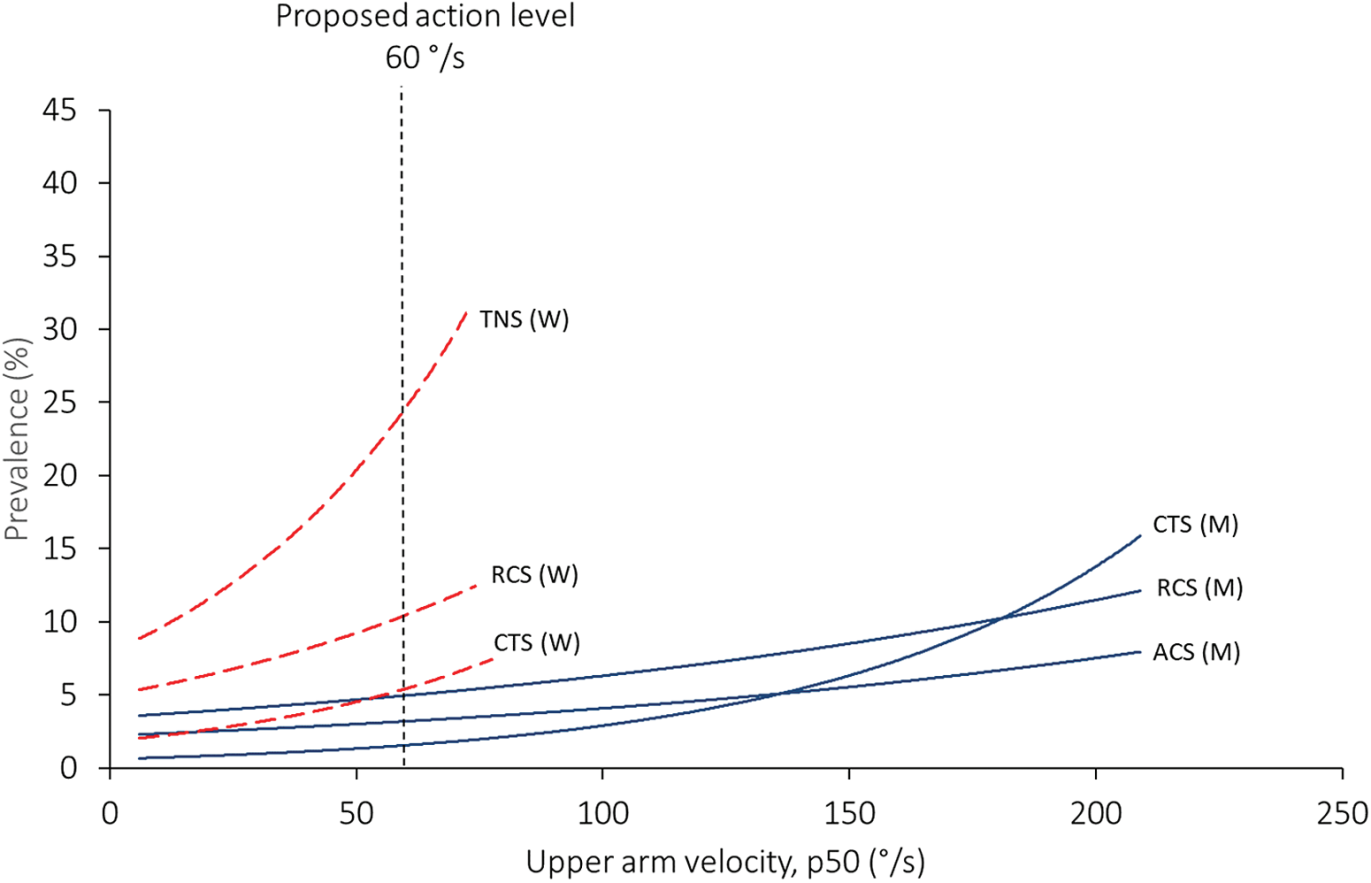
Statistically significant associations between tension neck syndrome (TNS), rotator cuff syndrome (RCS), acromioclavicular syndrome (ACS), and carpal tunnel syndrome (CTS), versus upper arm velocity, in women (red dotted lines) and men (blue continuous lines), calculated by Poisson regression. Modified from Balogh *et al.* (2019).

In most cases, the action levels were defined based on the calculated exposure–response relationships. We could not identify any obvious thresholds at which the risk of MSDs starts to increase in any of the displayed exposure–response relationships. The risk of MSDs increased monotonously with increasing exposure, and several of the diagnosed disorders were more than twice as common in groups with high wrist- or upper arm velocity, than in those with low (Figs 1 and 2). Thus, one may raise the question of how to determine specific values of the action levels. How many individuals with MSDs should we accept? There are no obvious answers to these questions.

Concerning exposures where our studies have shown a statistically significant relationship between exposure and MSDs, we have chosen to set the action levels so that the groups of workers who exceeded the action levels in our dataset showed a high prevalence of MSDs. For a few, we did not show such relationships but they have been clearly demonstrated by other researcher (see below). Then, we set the action level based on the literature.

There are no guarantees that loads below the action levels are free of risk. There is variation in individual sensitivity, which means that some individuals are at risk of harm at lower levels. Furthermore, some individuals might be exposed to too high load even though the group average is below the action level. Also, the proposed action level may be too high. Thus, the specific values of the action levels are open for discussion and may be revised. The proposed action levels for physical workload are given in Table 1. The suggested action levels are independent of working environment, sex, age, and other personal circumstances, as is common for OELs.

**Table 1.** Proposed action levels for physical workload concerning movement velocities, postures, muscular load and time for recovery.

	Proposed action levels			
	10th percentile^a^	50th percentile^a^ (median load)	90th percentile^a^ (peak load)	Time for recovery ^b^
				
*Movement velocity*				
Upper arm	-	60°/s	-	-
Wrist^c^	-	20°/s	-	-
*Posture*				
Head extension/flexion	-10°	< 0° or >25°	50°	-
Elevated upper arm^d,e^	-	30°	60°	-
*Muscular load*				
Trapezius muscle	-	-	20% MVE	5% of time
Forearm extensor muscles	-	10% MVE	30% MVE	5% of time

^a^ High risk of disorders at higher exposure.

^b^ Proportion of time with muscular activity <0.5% MVE. High risk of disorders with shorter time for recovery.

^c^ If the work is also force demanding, the suggested action level is 15°/s.

^d^ Elevation in relation to the vertical.

^e^ Only applicable if the arms are not supported (e.g. on a table or other surface).

## Considerations

Several of the exposure–response relationships that were reported in our studies concerned wrist movements. Fig. 1 shows all statistically significant exposure–response relationships between wrist velocity and diagnosed disorders (Balogh *et al.*, 2019). Many relationships were found between wrist velocity and conditions involving MSDs in the shoulder and neck, most likely because high wrist velocities usually occur simultaneously with other harmful exposures. By applying the equations provided by Poisson regression in Balogh *et al.* (2019) we have estimated possible reductions in prevalence of disorders by applying the suggested action levels. If the work load in the male group that was exposed to the highest wrist velocities in our dataset was reduced from 55°/s to 20°/s, the prevalence of CTS may be reduced by from 18% to 1%, which is a reduction by 93% (Fig. 1). Correspondingly, by reducing upper arm velocity in the highest exposed female group from 73°/s to the suggested action level 60°/s, the prevalence of pronounced neck/shoulder myalgia with clinical findings (tension neck syndrome) could be reduced from 32% to 25%, i.e. a reduction by 22% (Fig. 2). In the highest exposed male group (upper arm velocity 209°/s) the prevalence of rotator cuff syndrome would be reduced by 59%, from 12% to 5%.

Concerning other exposures, e.g. in female workers the prevalence of tension neck syndrome was about 25% in the quintile with the highest peak load in the trapezius muscle, while it was about 10% in the quintile with the lowest, rendering a relative risk of 2.5 (Balogh *et al.,* 2019). The muscular load in the trapezius was also statistically significantly associated with several other diagnosed disorders. We chose to define an action level only for the peak load of trapezius activity, which we consider to be clearly associated with the physical workload, while the median load may to a higher extent be affected by personal- and stress-related factors.

Despite the high number of technical measurements in our studies, there was insufficient data for certain exposures. We could not identify statistically significant associations between MSDs and some of the well-established ergonomic risk factors. However, relationships have been demonstrated between prolonged neck extension and neck pain (e.g. Takamiya *et al.*, 2006; van den Heuvel *et al*., 2007), neck flexion and neck pain with physical findings (Palmer and Smedley, 2007), work with elevated arms and disorders of the shoulders (e.g. Bodin *et al.*, 2012; Dalbøge *et al.*, 2014, 2018), and lack of recovery in trapezius and MSDs (e.g. Veiersted *et al.*, 1993; Hanvold *et al.*, 2012, 2013). Thus, the proposed action levels for neck extension, elevated arms and lack of recovery in trapezius were based on knowledge from other scientific literature. Most likely, the reason for not finding these associations in our studies was because we have included only a few occupational groups who were exposed to those risk factors.

The action levels were determined for one exposure at the time. However, it is even more relevant to define action levels for combination of exposures. It has been shown that the risk increases when for example, repetitive work is combined with high loads and/or performed using vibrating tools (van Rijn *et al.*, 2009). Such an approach is the TLV for hand activity (ACGIH, 2019), which is based on observations of hand movements and estimates of applied force. As a start, we reduced the action level for wrist velocity from 20°/s to 15°/s if the work is also high-force-demanding and/or is performed using vibrating tools.

## Implementation

Large efforts are made by researchers and commercial stakeholders to simplify the methods for quantitative measurements, and the equipment needed to collect and analyze the data (Dalbøge *et al.*, 2014; Dahlqvist *et al.*, 2018). Furthermore, within the EU, a project is ongoing to harmonize technical methods of measurement: “PEROSH Recommendations for Procedures to Measure Occupational Physical Activity and Workload” (Perosh, 2018).

Through training programs (1–2 days) directed to professionals in the work environment field, such as personnel in occupational health service, work protection authorities and local safety representatives, it is possible to build up a competence that can be used for recordings of the physical workload within their affiliated companies and organizations. The training programs should include the methodology for practical measurements in the field, measurement strategies, and interpretation of results.

Risk assessment based on technical recordings and action levels are well suited for integration in ergonomic programs and systematic occupational health efforts. By measuring the load throughout the working day, you can identify ergonomic risk factors and the specific tasks in which they occur. This provides a basis for prioritizing preventive actions in terms of technical/practical interventions and/or organizational improvements, e.g. by organizing a job rotation that ensure that the workload during the day falls below the action levels. To report the exposure in quantitative terms and relate it to the proposed action levels facilitates the communication with for example joint ergonomics committees and production engineers.

Technical recordings may be combined with observations. The former provides objective data on exposures which could be difficult or even impossible to obtain by observations alone, such as velocities. Complementary observational methods may be needed, e.g. in risk assessment of short-term heavy lifting or high-force-demanding tasks, which can themselves present a risk of injury. Such short-term high loads may be “diluted” when technical recordings are made over a complete working day.

## Conclusions

We propose several action levels for physical workload, based on associations between technical recordings of the exposure and myalgia, tendon disorders and nerve entrapments in the upper musculoskeletal system. For example, we propose that wrist velocity as a median over an 8-hour workday should not exceed 20°/s. Our ambition is to spread these action levels and to reach a consensus on standardized limits for physical workloads. The long-term goal is that OELs shall be introduced in legislation. We are aware that the action levels presented may need to be revised. We hope that quantitative measurements, combined with action levels, will become an integral part of systematic occupational health efforts, enabling reduction and prevention of work-related MSDs.

## Abbreviations

CTS, carpal tunnel syndrome; MSD, musculoskeletal disorders; MVE, maximal voluntary electrical activity; OELs, occupational exposure limits; TLV, threshold limit values
